# Adult Manifestation of Lower Urinary Tract Dysfunction as Daytime Urinary Incontinence and Nocturnal Enuresis in a Case With Spinal Lipoma

**DOI:** 10.1002/iju5.70032

**Published:** 2025-04-23

**Authors:** Taiju Hyuga, Koki Sugimura, Kimihiko Moriya

**Affiliations:** ^1^ Department of Pediatric Urology Jichi Medical University, Children's Medical Center Tochigi Shimotsuke Japan

**Keywords:** adult, lower urinary tract symptoms, Spina bifida

## Abstract

**Introduction:**

Generally, lower urinary tract function is considered to show few changes in adulthood for cases of spina bifida.

**Case Presentation:**

The case involved a 33‐year‐old man with a primary diagnosis of spinal lipoma. Urological management by spontaneous voiding was maintained, and uroflowmetry at 23 years old showed a maximum flow rate of 9.6 mL/s and a residual urine volume of 35 mL; then urological follow‐up was ended. The patient developed nocturnal enuresis and daytime urinary incontinence. Residual urine was exceeding 500 mL. Bladder deformity was identified on VCUG. UDS showed a high storage pressure with compliance of 5.0 mL/cmH_2_O. CIC management was introduced, and vibegron was initiated. After that, the urinary symptoms were resolved immediately. On video‐UDS, bladder deformity and compliance had improved.

**Conclusion:**

Patients managed by spontaneous voiding should be carefully evaluated for atypical UDS findings to decide whether urological follow‐up can be considered complete.

**Summary**

- We described a case of spinal lipoma in which LUTD manifested as daytime urinary incontinence and nocturnal enuresis after urological follow‐up ended upon reaching adulthood.
- Further examinations were performed, and urological managements need to be changed. After that, urinary symptoms and lower urinary tract function were improved.
- Patients managed by spontaneous voiding should be carefully evaluated for atypical UDS findings to decide whether urological follow‐up can be considered complete.

**Abbreviations**
CIC: clean intermittent catheterization
ICS: International Continence Society
LUTD: lower urinary tract dysfunction
LUTS: lower urinary tract symptoms
UDS: urodynamic study
VCUG: voiding cystourethrogram
VUR: vesicoureteral reflux

## 1 Introduction

In cases of spina bifida, the risk of decreasing lower urinary tract function due to spinal cord tethering increases during childhood and adolescence [1, 2]. Generally, lower urinary tract function is considered to show few changes in adulthood, and urological follow‐up is often ended for cases of spina bifida that do not have LUTS or LUTD in adulthood. In this report, we describe a case of spinal lipoma in which LUTD manifested as daytime urinary incontinence and nocturnal enuresis after urological follow‐up ended upon reaching adulthood.

## 2 Case

**FIGURE 1:**
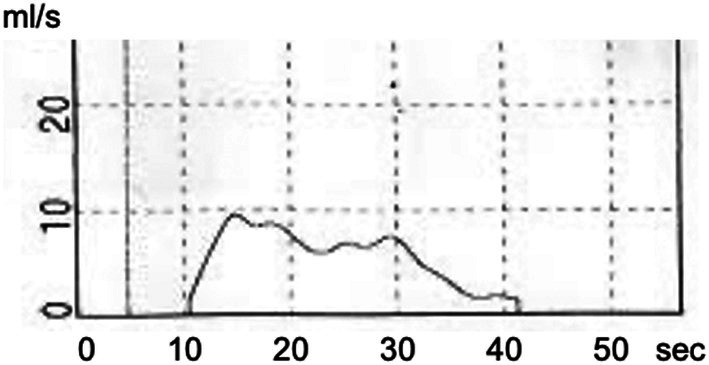
Uroflowmetry at the end of regular hospital visits (23 years old). The flow pattern was not a typical bell pattern. Maximum and average flow rates were 9.6 and 5.8 mL/s, respectively. Post‐void residual urine was 35 mL.

The case involved a 33‐year‐old man with a primary diagnosis of spinal lipoma. Resection surgery had been performed for the spinal lipoma at 1 month. After the surgery, lower urinary tract management by spontaneous voiding was performed at a children's hospital, mainly using ultrasound. At 19 years old, the patient was transitioned to the general urology department, where he was managed using uroflowmetry. Management by spontaneous voiding was maintained, and uroflowmetry at 23 years old showed a maximum flow rate of 9.6 mL/s and a residual urine volume of 35 mL (Figure 1). Since LUTS was not observed, urological follow‐up was ended.

**FIGURE 2:**
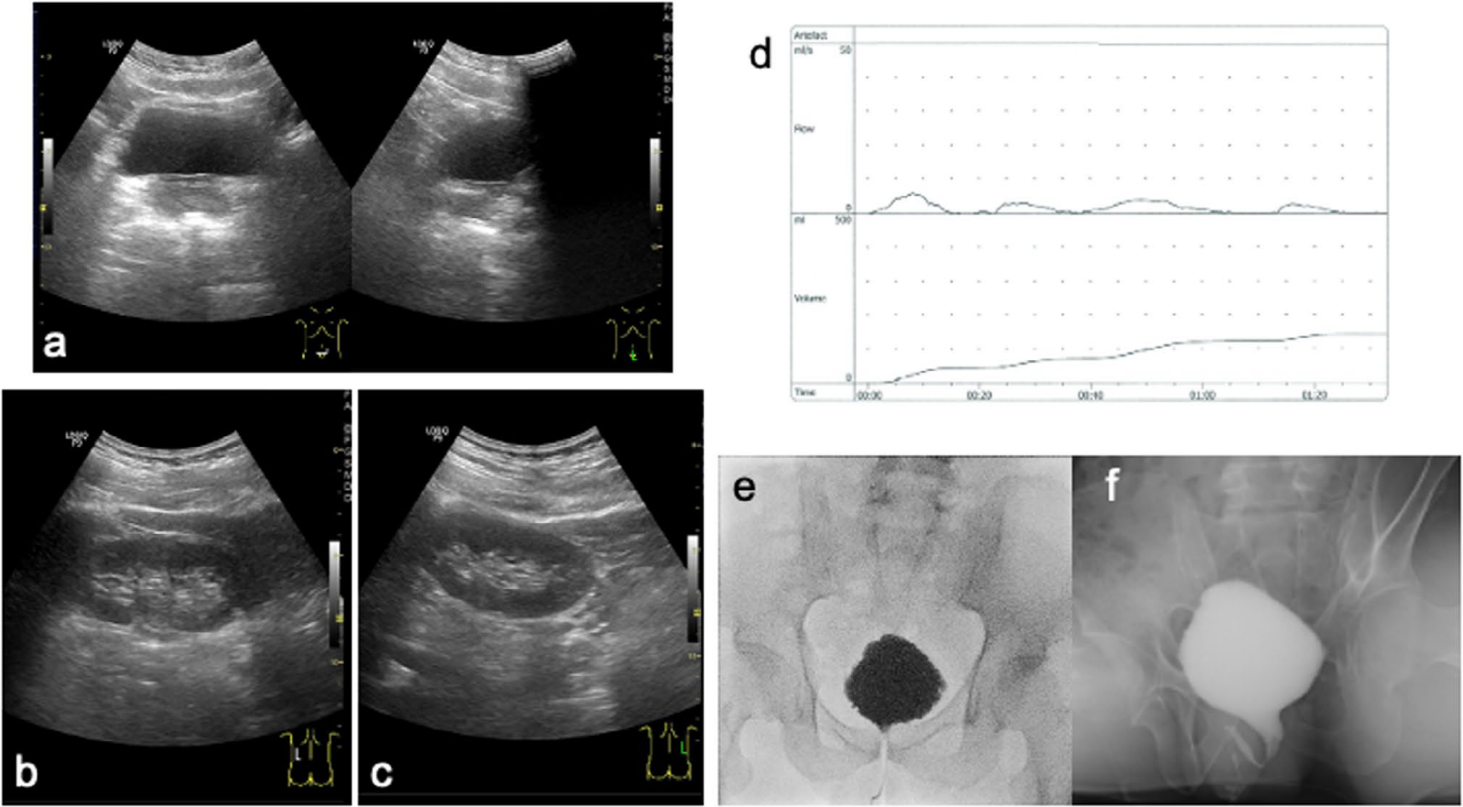
Abdominal ultrasonography (a–c), uroflowmetry (d), and VCUG (e, f). (a) Bladder. No bladder deformity is apparent. (b) Right kidney; (c) left kidney. No dilatation of either upper urinary tract is evident. (d) Findings from uroflowmetry. Voided volume, 142 mL: Residual urine volume, 550 mL; maximum flow rate, 5.9 mL/s; flow pattern, interrupted. (e) VCUG in storage phase. Bladder deformity is revealed, VUR is not present. (f) VCUG in voiding phase. Relaxation of the urethral sphincter is inadequate.

**FIGURE 3:**
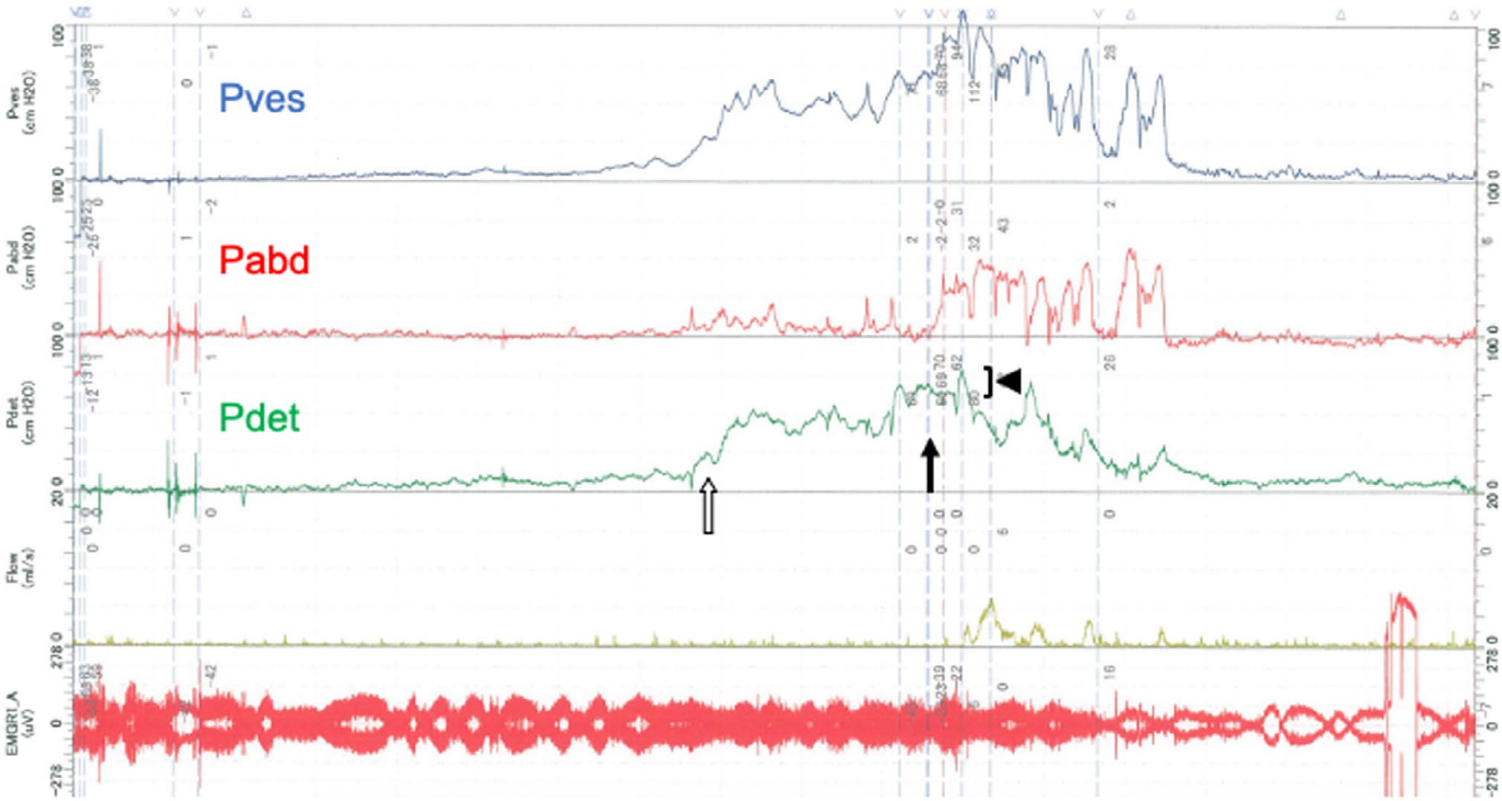
Urodynamic study (pressure flow study). The infusion volume at first desire to void is 125 mL, at which time detrusor pressure has already increased to 25 cmH_2_O (white arrow). The volume at maximum desire to void is 180 mL, with detrusor pressure of 70 cmH_2_O (arrow). In the voiding phase, straining to void is present. Detrusor pressure is 10 cmH_2_O (arrowhead), suggesting detrusor underactivity.

**FIGURE 4:**
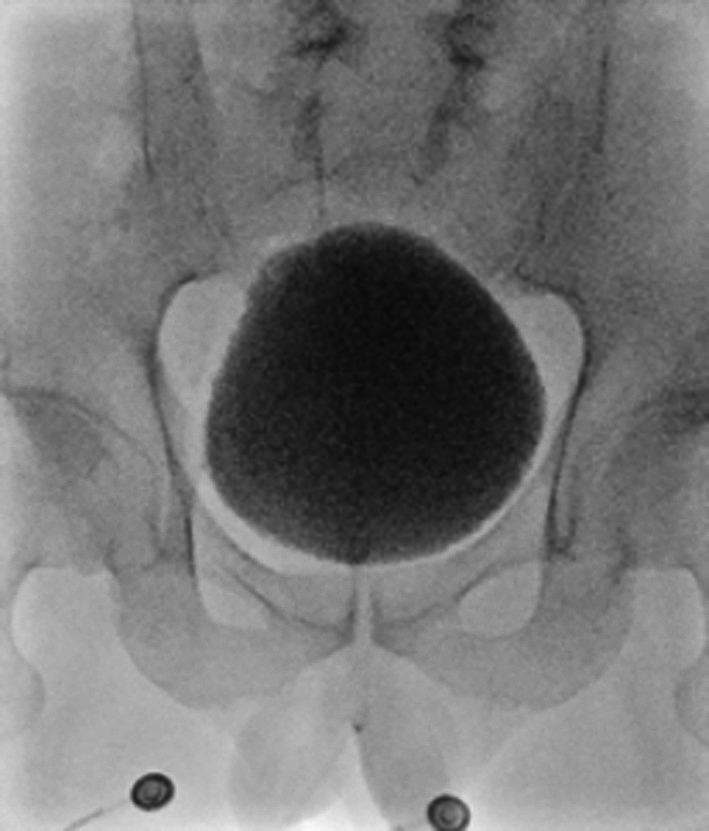
Video‐UDS. No bladder deformity is evident and shape of the bladder has improved.

At 31 and 32 years old, the patient developed nocturnal enuresis and daytime urinary incontinence. He complained of these symptoms and was referred to our department. The value of serum creatinine was in the normal range (0.65 mg/dL) at 27 years old. There was no episode of urinary tract infection and no symptoms of defecation. Any worsening of spinal cord tethering in MRI was not shown compared to 3 years ago. An abdominal ultrasound showed no bladder deformity or dilation of the upper urinary tract (Figure 2a–c). Uroflowmetry showed an interrupted pattern and residual urine exceeding 500 mL (Figure 2d). Bladder deformity was identified during the VCUG storage phase, and incomplete relaxation of the urethral sphincter was revealed during the voiding phase. No VUR was observed (Figure 2e,f). UDS showed a high storage pressure with compliance of 5.0 (125/25) mL/cmH_2_O. During the voiding phase, the voiding pattern showed straining to void, and an increase in detrusor pressure of 10 cmH_2_O. showed detrusor underactivity (Figure 3). Regarding lower urinary tract management, CIC management six times a day was introduced and vibegron was initiated at 50 mg/day. After changing the lower urinary tract management, daytime urinary incontinence and nocturnal enuresis were resolved immediately. On video‐UDS 6 months after starting CIC, bladder deformity had improved and compliance had improved to 11.7 (350/30) mL/cmH_2_O (Figure 4).

## 3 Discussion

In this case maintained by spontaneous voiding, lower urinary tract function changed through adolescence to adulthood. Lower urinary tract function deteriorated during adulthood, forcing a change in management. Lower urinary tract function is generally considered to show little change in adulthood [2].

However, some reports have described cases with deterioration of lower urinary tract function in adulthood [3, 4, 5, 6]. Chan et al. reported that half of the 24 cases of adult spina bifida (age range: 16–27 years) showed deterioration of lower urinary tract function, and that additional treatment such as bladder augmentation or botulinum toxin injection therapy was required [7]. One report followed lower urinary tract management for 5250 cases of spina bifida from childhood to adulthood. Of those, 20.0% of cases were managed by spontaneous voiding in childhood, 15.4% in adolescence, and 10.5% in adulthood, showing a decrease in the number of cases managed by spontaneous voiding [8]. Those reports found that a certain proportion of cases changed urinary management through adolescence to adulthood because of lower urinary tract dysfunction and other reasons.

Actually, lower urinary tract management in this case was being performed by spontaneous voiding, and follow up had already been ended. As a result, the deterioration of lower urinary tract function was not noticed over the long term and was only recognized when the patient became aware of his own symptoms. Whether the lower urinary tract dysfunction could have been predicted in follow up is unclear. LUTS were not observed at the end of follow up. UFM was performed frequently as a follow up tool.

According to reports from ICS, no consensus has been reached on the definition of abnormal residual urine volume in adults [9]. Clinically, < 30 mL is not thought to be significant residual urine, but attention is required if residual urine of 50 mL or more is always present [10]. In the present case, the residual urine measurement could not predict the prognosis, and this may be said to be the limit of residual urine measurement.

Maximum urine flow rate in uroflowmetry was clearly low, at < 10 mL/s, which would be unusual even in a case like the present one [11, 12]. We suggest that follow up may be required for cases with abnormal findings from UFM. In addition, invasive UDS might be effective for evaluating lower urinary tract function if UFM shows atypical findings [13]. Regarding follow‐up, since bladder deformity could not be detected using ultrasound alone in this case, it is recommended that UDS be kept in mind for follow up tool. It is also important to inform patients of the risk of deterioration of lower urinary tract function in adulthood and to receive information if there are any changes.

## 4 Conclusion

Spina bifida patients with atypical findings on examination should be aware of the risk of deterioration of lower urinary tract function in adulthood. In particular, patients managed by spontaneous voiding should be carefully evaluated for atypical UDS findings or symptoms, to decide whether urological follow‐up can be considered complete. Pediatric urologists, adult urologists, and physicians involved in transitional care need to communicate together to ensure a smooth transition, and all should be aware that there are cases that require treatment in adulthood.

## Ethics Statement

The authors have nothing to report.

## Consent

Informed consent was obtained from the patient.

## Conflicts of Interest

The authors declare no conflicts of interest.
